# Comment on “Workplace Bullying as a Risk Factor for Musculoskeletal Disorders: The Mediating Role of Job-Related Psychological Strain”

**DOI:** 10.1155/2016/7843106

**Published:** 2016-08-17

**Authors:** Pracheth Raghuveer

**Affiliations:** Department of Community Medicine, Yenepoya Medical College, Yenepoya University, Mangaluru, Karnataka 575018, India

Here is a letter on the article published by Vignoli et al. on “Workplace Bullying as a Risk Factor for Musculoskeletal Disorders: The Mediating Role of Job-Related Psychological Strain” [1]. This study focuses on bullying in the workplace and its relationship with musculoskeletal disorders (MSDs). I happened to read the aforementioned article out of curiosity, and I must admit that the authors have put in a novel effort to investigate the problem.

Workplace bullying is regarded as a grave issue in the society today. This problem is further compounded by the dearth of proper assessment tools for research, as reported by Cowiea et al. [2]. The scientific background and rationale for the investigation have been clearly mentioned by the authors in the introduction. What makes this study unique is the fact that this study is one of the very few studies which highlight the nonpsychological effects of bullying at workplace [3, 4]. Furthermore, another distinctive feature of this article is the authors' attempt to control the potential confounding variables like age, gender, organizational role, type of contract, and physical demands. I totally agree with the authors when they state that the usual way of assessing workplace bullying by enquiring from the respondents if they feel that they were victimized as a result of bullying may lead to an element of subjective bias. Thus, the questionnaire based assessment confers a sense of objectivity to this study. Additionally, the authors also probed the role of job-strain as a mediator for MSDs, the analysis of which showed that job-related strain mediates the relationship between bullying and all MSDs, MSDs of the shoulders being a notable exception [1].

It is strongly recommended to use the Strengthening the Reporting of Observational Studies in Epidemiology (STROBE) Checklist while reporting cross-sectional studies [5]. When I critically reviewed this article, I observed that the authors have by and large adhered to the STROBE Checklist, which is commendable. Moreover, the authors have expressed the results in the form of 95% confidence intervals, in addition to *p* values. It is highly recommended to use both of these statistical measures in a research article, as they provide complementary information [6].

However, there are a few issues which need to be addressed. Certain serious consequences of workplace bullying which include cardiovascular disease [7] have been neglected by the authors. Workers who have been subjected to bullying are likely to have a 2.3 higher odds of cardiovascular disease, when compared to nonbullied workers, as reported by Kivimäki et al. [7]. This study would have been more valuable had the authors made an attempt to explore the relationship between workplace bullying and other important medical consequences in the study population, rather than only confining themselves to MSDs. Moreover, it has been reported that workplace bullying leads to alarming socioeconomic consequences too, which includes issues like sickness absenteeism and loss of employment. A study of these socioeconomic effects would have certainly added more weightage to this research. A study conducted in a sample of Italian workers revealed higher odds for sickness absenteeism for bullied workers when compared to those not exposed to workplace bullying (men: Odds Ratio = 1.62; women: Odds Ratio = 2.15) [8].

Besides, there is no mention of the study period in the article. According to the STROBE Checklist, it is important to describe the relevant dates, which includes the period of data collection [5]. Furthermore, the authors should have mentioned if they had obtained clearance from the Ethics Committee of their institution, prior to data collection. All studies must be approved by the Ethics Committee before starting, the detailed information of which should be provided in the manuscripts [9].

Nevertheless, this study demonstrates the relationship between workplace bullying and MSDs, with job-strain acting as a mediator. Thus, the authors deserve adulation for their exhaustive work.
